# Endovascular treatment of postoperative aortic coarctation aneurysms—a single center experience

**DOI:** 10.3389/fcvm.2024.1441867

**Published:** 2024-12-23

**Authors:** Iva N. Dimitrova, Peyo Simeonov

**Affiliations:** Department of Cardiology, University Hospital ’St. Ekaterina’, Medical University of Sofia, Sofia, Bulgaria

**Keywords:** aortic coarctation aneurysms, endovascular treatment, surgical repair, single center, imaging surveillance

## Abstract

**Background:**

Formation of local type aortic aneurysm years after surgical repair of coarctation (CoA) occurs in 10% of patients independent of the surgical technique and is a potentially life-threatening condition if left untreated with a high risk of aortic rupture. Redo open surgery is associated with 14% in-hospital mortality and a high risk of complications. Endovascular treatment appears to be a feasible alternative with a high success rate and low morbidity and mortality, but data concerning long-term results is still mandatory. We describe the single center experience of a series of patients treated with endovascular stent grafting for large aneurysms after previous surgery for CoA.

**Methods:**

This series involves 12 consecutive patients treated with endovascular stent grafting from April 2003 to January 2022 for late aneurysm at the site of previous surgical repair for CoA. Data on baseline characteristics, clinical, computed tomography (CT), procedural features, and in-hospital and long-term results were analyzed. All patients signed institutional informed consent for the procedure.

**Results:**

A total of 12 patients (average age 38.5 ± 10.2 years) underwent endovascular repair. The average interval between the initial surgical intervention and the aneurysm repair was 24.1 ± 9.1 years and the majority (83.3%) underwent synthetic patch aortoplasty as previous intervention. All patients were symptomatic at presentation with an average maximum diameter of the aneurysm 67.2 mm (ranging from 44 to 110 mm). Stent-graft placement was successful in all cases without procedural, in-hospital, 30-day, and one-year mortality or major complications. The average hospital stay was 9.3 days (range 7–19 days). For a mean follow-up period of 87 months (range, 23–168 months), all patients demonstrated positive aneurysm remodeling with cavity thrombosis and aneurysm diameter reduction to 54.9 mm (±16,6). For the entire follow-up period, we observed one aneurysm-related death and three deaths of heart failure.

**Conclusion:**

Endovascular stent-graft treatment of patients with thoracic aneurysms after repair of CoA is an acceptable treatment of choice. It has shown promising results with high technical success and low immediate, short—and mid-term morbidity and mortality. Still, randomised control trials (RCTs) are needed to define the long-term outcome of this approach. Routine surveillance and screening of patients with previous CoA repair are mandatory.

## 1 Introduction

Coarctation of the aorta (CoA) is a common congenital heart defect (CHD), accounting for 5%–8% of CHDs and a frequency of 4 per 10,000 live births with male predominance (1). It represents a narrowing of the aorta, with typical localization usually in the region of the ductus arteriosus, distal to the left subclavian artery (LSA). It can be associated with coexisting CHDs as a bicuspid aortic valve (BAV), aortic arch hypoplasia and other arch anomalies, ventricular septal defect (VSD), mitral valve abnormalities, subaortic stenosis (2). The prognosis of unrepaired CoA is not benign; authors report an average survival rate of 35 years of age (3).

Surgical repair is standard treatment in children and adolescents. Regardless of the advancements in surgical techniques and the high success rate, the prognosis of patients who have undergone CoA repair is not so favorable compared to that of age-matched counterparts in the general population, with only 65% of patients surviving to the age of 70 (4). Late post-repair complications occur decades after surgery and may require reintervention. The most common long-term cardiovascular complications after CoA repair of note are recoarctation (3%–41%), aortic aneurysm of the ascending aorta or at the site of previous coarctation (3%–38%), and systemic hypertension (25%–62%) (1, 2, 5, 6).

Aortic aneurysms at the site of the repair occur in 10% of the patients in the long-term follow-up and are potentially life-threatening if left untreated with a high risk of aortic rupture (6). Patch grafts and advanced age (above 13.5 years) at the index coarctation repair are independent predictors of aneurysm formation at the site of previous coarctation repair (6). Subsequent management of local type aneurysms after initial CoA repair is a challenge, and repeat surgery is associated with 14% in-hospital mortality and high risk of paraplegia, paralysis of recurrent laryngeal nerve, and bleeding (7).

Endovascular treatment appears a feasible alternative to redo open surgery for post-repair complications. Although small series are reported, they show encouraging results- a high success rate- of up to 100% and a low rate of complications and mortality (8). On the other hand, thoracic endovascular aortic repair (TEVAR) is less invasive and requires a short hospital stay. Data concerning long-term results is mandatory to support the advantages of the endovascular approach in treatment of late thoracic aortic aneurysms (TAA) after surgical repair of CoA over surgical re-intervention.

We aimed to represent the single center experience of endovascular aortic repair for local type thoracic aneurysms after previous surgery for CoA.

## 2 Material and methods

The database of patients with thoracic aneurysms after open surgical aortic CoA repair (n-12) treated with endovascular aortic stent grafts at University Hospital “ St. Ekaterina” - Sofia from April 2003 to January 2022 was reviewed. They constitute 42.9% of all patients who underwent TEVAR for thoracic aortic aneurysms. Information concerning baseline characteristics, initial surgical repair, concomitant CHD, clinical, radiographic, and procedural features, and in-hospital and long-term outcomes were retrospectively obtained from the hospital electronic database, including medical reports, procedure protocols, imaging examination protocols, discharge documents, and outpatient clinic reports. All patients had follow-ups in our medical institutions, and data were collected through visits, medical charts, phone calls, and postprocedure computed tomography angiography (CTA) examinations. All patients signed institutional informed consent forms for the procedure.

### 2.1 Indications for therapeutic endovascular procedure

The indications for intervention are based on the existing current recommendations for the treatment of patients with thoracic aortic aneurysms: For asymptomatic, uncomplicated aneurysms when the diameter is ≥55 mm; in the presence of risk factors for aortic rupture as -rapid growth of the aorta (≥0.5 cm/year); symptomatic aneurysms (presenting with pain, bronchial compression leading to dyspnea or hemoptysis, compressive lung atelectasis, hemothorax, hemomediastinum, dysphonia, esophageal compression with dysphagia, compression of the main branch of the pulmonary artery mimicking pulmonary embolism); underlying connective tissue disorder or hereditary aortopathy; morphology of a saccular aneurysm; female gender; infected aneurysm or complicated aneurysms (ruptured or with impending rupture) a lower aortic size threshold (<55 mm) is considered (9).

It is important to note that all patients (n-12) in the current series were symptomatic and are considered as exhibiting high-risk features.

### 2.2 Imaging modality

Computed tomography (CT) is established as the gold standard for diagnosis, intervention planning, and follow-up of treatment outcomes in patients with aortic diseases. It allows the visualization of the entire aorta and its branches with high spatial resolution and the quick acquisition of three-dimensional reconstructions of TAA. All patients underwent contrast-enhanced pre-procedural CTA. We used electrocardiogram-gating in order to minimize the motion artifacts. The study was done by a contrast-enhanced scan after intravenous infusion of 50–80 ml Iodine-containing contrast medium at a rate of 4 ml/s, using a Multi-Detector Computed Tomography (MDCT). The cross-section images, multiplanar, and volume reconstructions were the basis for procedure planning.

All patients underwent control CTA before discharge. A follow-up CTA is performed at 1 month, and if the findings are suitable (well-positioned stent graft, no fractures or compression, no endoleaks, completely isolated and thrombosed aneurysm, patent visceral branches without evidence of malperfusion, no stent graft migration, and clinically asymptomatic patient), a follow-up CTA is conducted after 1 year and annually thereafter. CTA was performed using the Toshiba Aquilion ONE 320-slice CT Scanner.

### 2.3 Endovascular stent-grafts

For aneurysm exclusion, four types of thoracic endograft were used: Gore TAG – endoprosthesis (W. L. Gore, Sunnyvale, Calif, USA) in 5 patients; Relay – Thoracic Stent-Graft (Bolton Medical, Inc.-USA/Terumo Aortic) in 4 patients; Zenith Th2 and Zenith Alpha (COOK medical) in 2 patients and EndoFit (LeMaitre Vascular) in 1 patient. Gore TAG – endoprosthesis (W. L. Gore, Sunnyvale, Calif, USA) is an expanded polytetrafluoroethylene (ePTFE) graft with a self-expanding nitinol support structure. It is delivered through an 18F to 24F sheath. Relay – Thoracic Stent-Graft comprises self-expanding nitinol stents sutured to polyester fabric graft. The profile of the primary introducer sheath ranges from 22 to 26F. Zenith TX2 grafts are made from stainless steel Z-stents attached to a woven polyester fabric in a two-piece design. The device requires a 20F to 22F delivery system and Zenith Alpha – nitinol stent material attached to a woven polyester graft, requiring 16–20F sheath compatibility. EndoFit is made of a metallic nitinol skeleton, covered by a polytetrafluoroethylene membrane and delivery device from 22 to 24 F. The prosthesis's length is defined to cover all segments of the aneurysmal aorta. The various endografts require landing zones of different lengths according to their manufacturer, but the minimum required zone is 15 mm. The graft diameter is chosen by oversizing by up to 30% of the proximal landing zone's diameter to avoid type I endoleak. The choice of a specific еndovascular stent-grafts over another was, on one hand, dependent on the anatomical characteristics of the patient, determined during the pre-procedural planning based on the performed CT scan, with primary consideration given to the diameter of the landing zone and the available sizes offered by the manufacturer. On the other hand, the choice was made in accordance with the current national health insurance policy in our country.

### 2.4 Procedure

All 12 procedures were performed under general anesthesia with mechanical ventilation in a catheter lab with the collaboration of an interventional cardiologist, vascular surgeon, and anesthesiologist, and two of the interventions were accomplished immediately after aorto-carotid bypass. Arterial access-femoral (11 patients) or iliac (1 patient) was obtained using a vascular surgical approach. Heparin anticoagulation was used to maintain activated clotting time in desired ranges. A 5 Fr arterial introducer was placed in the left radial artery, through which a Pigtail catheter was advanced into the ascending aorta for performing aortography for an initial control to verify the placement of the guide wire/endoprosthesis, for control injections during positioning of the prosthesis in the precisely chosen landing zone, and as a marker for the ostium of the LSA and finally-for verification of the procedure result. A 6 Fr arterial introducer was placed in the femoral/iliac artery, and a 0.035-in guidewire was advanced to the aortic valve. The diagnostic guidewire was exchanged over the Judkins right catheter with a stiff wire (Amplatz Ultra-stiff by COOK Medical, Lunderquist Extra stiff Wire by COOK Medical), ensuring its position was maintained under fluoroscopic control. After confirming that the stiff wire was in the desired location, the endoprosthesis was placed over it at the desired position in the aorta. Visualizing the aortic arch and precisely positioning the endoprosthesis is typically done in the left anterior oblique projection (LAO 30°–40°), as this projection provides the best visualization of the aortic arch and its branches. During deployment, we typically used rapid pacing at 180 beats per minute to reduce systolic blood pressure to <60 mmHg to prevent stent graft migration and position it accurately in the desired zone. After deployment, a completion angiogram was performed.

The technical success rate, defined as the successful placement, positioning, and deployment of the endoprosthesis(es) and isolation from the bloodstream of the aortic aneurysms, was 100%.

### 2.5 Statistical analysis

Descriptive statistics - The quantitative variables are presented through summary statistical characteristics - mean, median, and standard deviation (SD). - The categorical variables are presented as absolute frequencies (n) and relative frequencies (%).

## 3 Results

The baseline characteristics of patients are listed in Table 1. All (N-12) patients had a history of previous surgical repair of CoA in their childhood or adolescence. The average age of aneurysm detection and, accordingly, of the conducted endovascular treatment was 38.5 ± 10.2 years; the youngest patient was 25y/o, the oldest- was 51y/o, and the average age of index surgical repair was 14.4 ± 5.4 years. The average interval between the initial surgical intervention and the TEVAR for aneurysm correction was 24.1 ± 9.1 years. Male gender was predominant, and 3/4 of patients had arterial hypertension (AH). The most common index surgical technique for CoA correction was a synthetic patch aortoplasty in 10 patients (83.3%). We did not have data on the presence of genetic syndromes in the patients, such as Turner syndrome, Noonan syndrome, etc. It is worth noting that all patients were symptomatic, with one patient presenting with contained aortic rupture, another with type B dissection, and seven with identified bronchial compression. In our country, there is a lack of established protocol for follow-up and surveillance of patients after correction of CoA, and the reason for re-presentation in all cases was the onset of symptoms.

**Table 1:** Baseline and clinical characteristics of patients.

Variable	*N* = 12
Mean age at endovascular repair (years ± SD)	38,5 ± 10,2
Male gender	9 (75%)
Risk factors	
AH	9 (75%)
Dyslipidemia	2 (16,6%)
DM	0
Smoking	6 (50%)
Concomitant CHD	
BAV	4 (33,3%)
ASD	2 (16,6%)
Age at index CoA repair (years ± SD)	14,4 ± 5,4
Time from index surgery to aneurysm detection (years ± SD)	24,1 ± 9,1
Type of prior CoA repair	
Patch angioplasty	10 (83,3%)
Subclavian flap	2 (16,7%)
Symptoms at admission	12 (100%)
Chest pain	5 (41,6%)
Dyspnea	5 (41,6%)
Hemoptysis	2 (16,7%)

AH-arterial hypertension; DM-diabetes mellitus; CHD, congenital heart defect; BAV, bicuspid aortic valve; ASD, atrial septal defect; CoA, coarctation of the aorta.

The initial findings from the CTA are presented in Table 2. The predominant morphology of TAA was saccular, observed in 8 patients (66.7%), considered to be at higher risk for rupture compared to fusiform shape, noted in 3 of our patients (25.0%). The average maximum diameter of aneurysm at presentation was 67.2 mm (range, 44–110 mm).

**Table 2:** Characteristics of the aortic pathological findings from the initial CTA.

Shape of aneurysm *N* (%)	
Fusiform	3 (25%)
Saccular	8 (66,7%)
Pseudoaneurysm	1 (8,3%)
Proximal lending zone diameter (mm ± SD)	24,9 ± 4,7
Size of aneurysm (mm ± SD)	67,2 ± 19,6
Length of the aneurysmal segment (mm ± SD)	94,1 ± 27,5
Distance from the subclavian artery (mm ± SD)	11,6 ± 12,3
Involvement of the subclavian artery *N*(%)	2 (16,7%)

None of the patients had CT data for intramural hematoma, pericardial, or pleural effusion.

**Figure 1:**
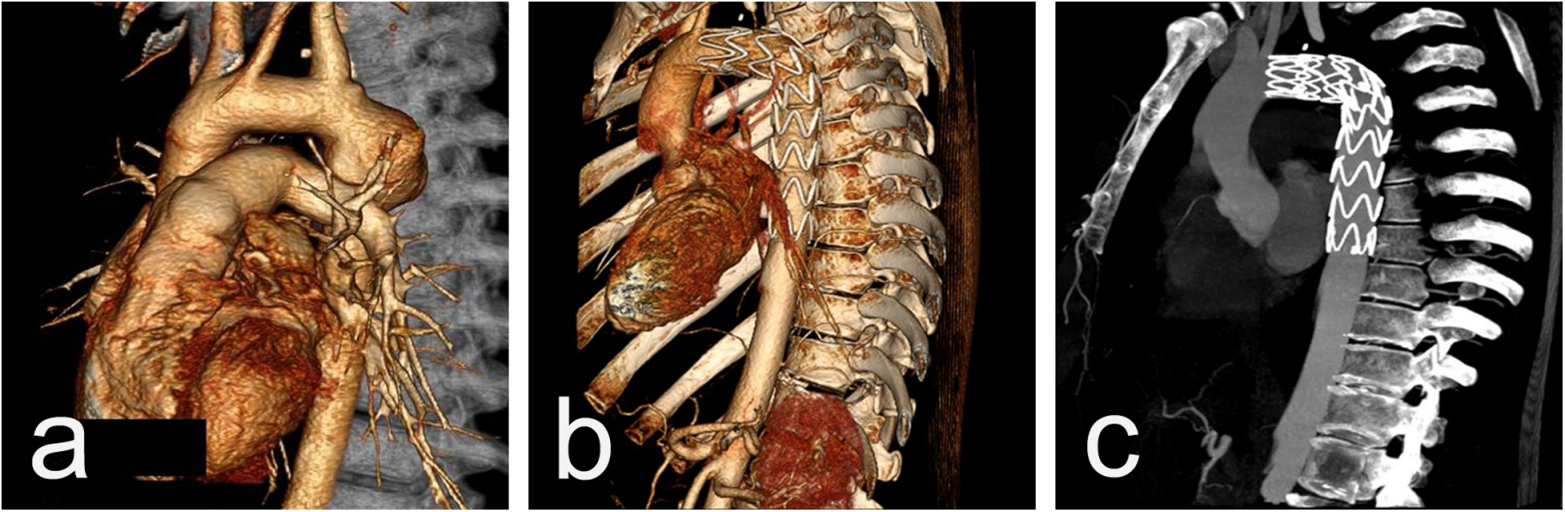
CT scan imaging before **(a)** and after **(b and c)** stent-graft implantation of 26y/o male presenting acutely with contained rupture of an aneurysm 20 years after initial CoA repair. The aortic aneurysm is located close to the origin of LSA **(a)** and the implanted graft excluded the aneurysmal sac but fully covered the LSA **(b)** with an excellent blood supply through the left vertebral artery from control CTAs **(c)**.

**Figure 2:**
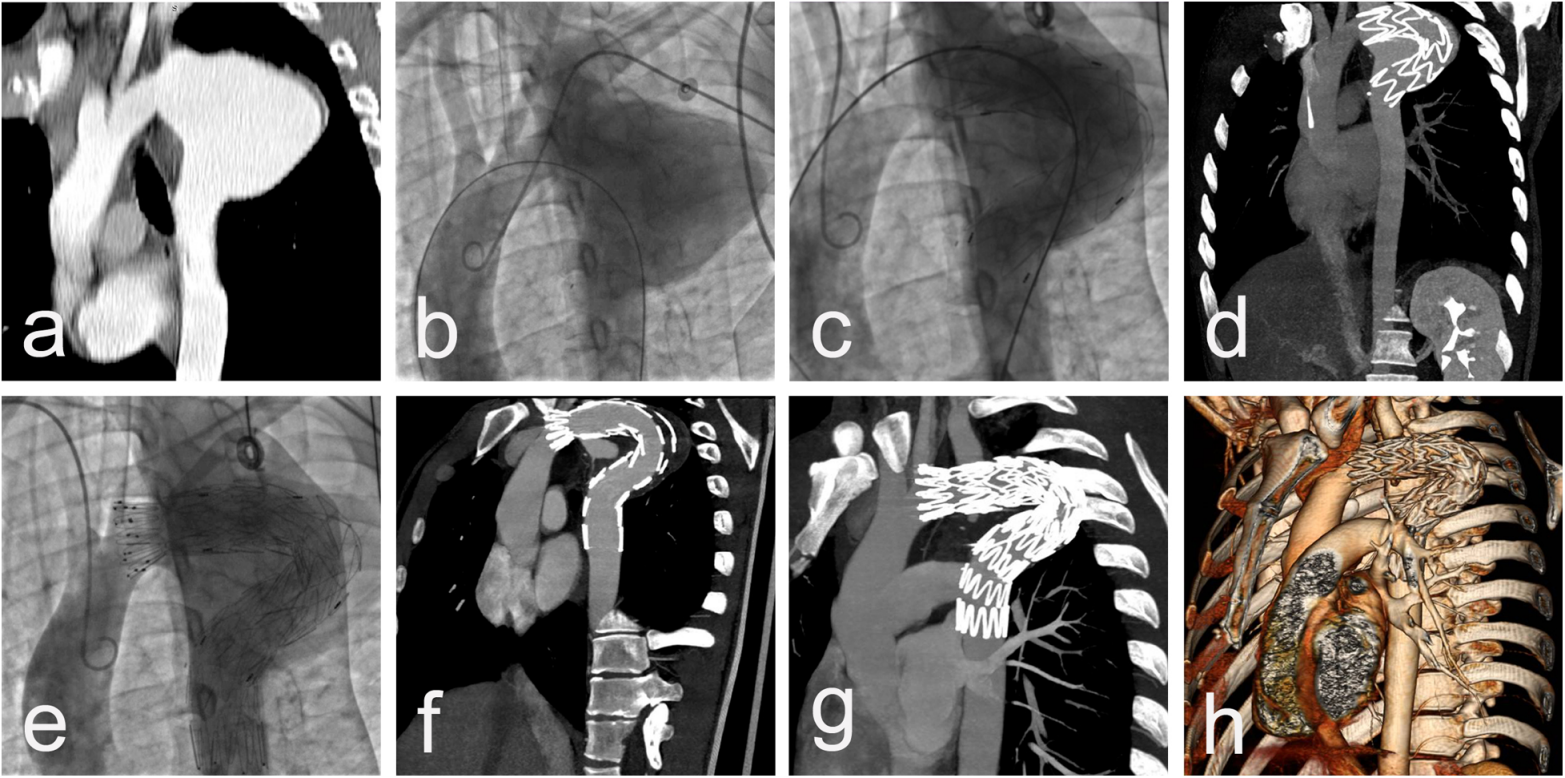
CT scan imaging **(a)** and aortic angiography **(b)** demonstrating saccular thoracic aortic aneurysm engaging LSA of 27 y/o male presenting 15 years after initial surgical repair with cough and hemoptysis. Dimensions of aneurysm 56/70 mm with no signs of thrombosis. Postdelopyment aortography revealing a collapse of the stent-graft in the large aneurysm, accepting its large curvature with severe type I endoleak **(c)**, confirmed by immediately performed CTA, with no sign of iatrogenic rupture and preserved distal blood flow **(d)** Additional stent-graft positioned right behind left carotid artery and complete aneurysm isolation **(e)** with post-procedure CT data of completely covered LSA and small endoleak type II from large LSA **(f)** CT scan imaging at 1-year follow-up with complete aneurysm thrombosis, reduction of the aneurysm size with 40% and lack of endoleaks **(g,h)**.

Procedural data: All cases were performed in a standard catheter lab environment. Stent-graft deployment was successful in all patients. In 5 patients, partial or complete coverage of the LSA was necessary to ensure an adequate landing zone for the endoprosthesis and to avoid endoleak. Two of them underwent aorto-carotid bypass to the left common carotid artery, performed prior to aortic stenting. No subclavian steal syndrome was observed during the hospital stay and subsequent follow-up (Figures 1a–c). Four patients required additional stent(s). Postdeployment aortography revealed type I endoleak in 4 patients, with one left under surveillance. while in the other three, additional endoprostheses were implanted to seal the leak (one received 2 additional endoprostheses, and the others received 1 endoprosthesis) with optimal results. In one of those patients, we observed a severe type I endoleak with a persistent filling of the aneurysm due to a collapse of the stent-graft (26/155 mm) in the large aneurysm, accepting its large curvature. A second endoprosthesis (28/200 mm) was positioned using two stiff guidewires to cross the old prosthesis. The additional stent-graft was positioned right behind the left carotid artery, and we achieved complete aneurysm isolation (Figures 2a–e). There was no procedural mortality and neurologic, vascular-access, or ischemic complications. All patients were cared for in the intensive care unit after the procedure, with a mean stay of 1.84 days. The average hospital stay was 9.3 days (7–19 days) (Table 3).

**Table 3:** Procedural details of the endovascular aneurysm repair and hospital stay.

Vascular approach *N*(%)	
Transfemoral	11 (91,7%)
Transiliac	1 (8,3%)
Total number of stent-grafts	18
Average stent-graft diameter (mm ± SD)	29,5 (4,3)
Average stent-graft length (mm ± SD)	163 (22,9)
Oversizing (%±SD)	18,5 ± 14,7%
Endoleak *N*(%)	
Type 1	4 (33,3%)
Length of intensive care unit stay, days	1,84 (1–3)
Hospital stay, days	9,3 (7–19)

During the hospital stay, the mortality was 0%, and we did not observe complications such as aneurysmal rupture, dissection, need for emergent surgical intervention for any cause, acute renal failure, neurological symptoms, or prolonged ventilation. Only one patient required hemotransfusion. All patients underwent control CTA before discharge. We observed the proper position of the stent grafts and completed aneurysm isolation in all patients. Endoleak type II from LSA was observed in 2 patients left under surveillance, and it was fully resolved within 6 months.

**Figure 3:**
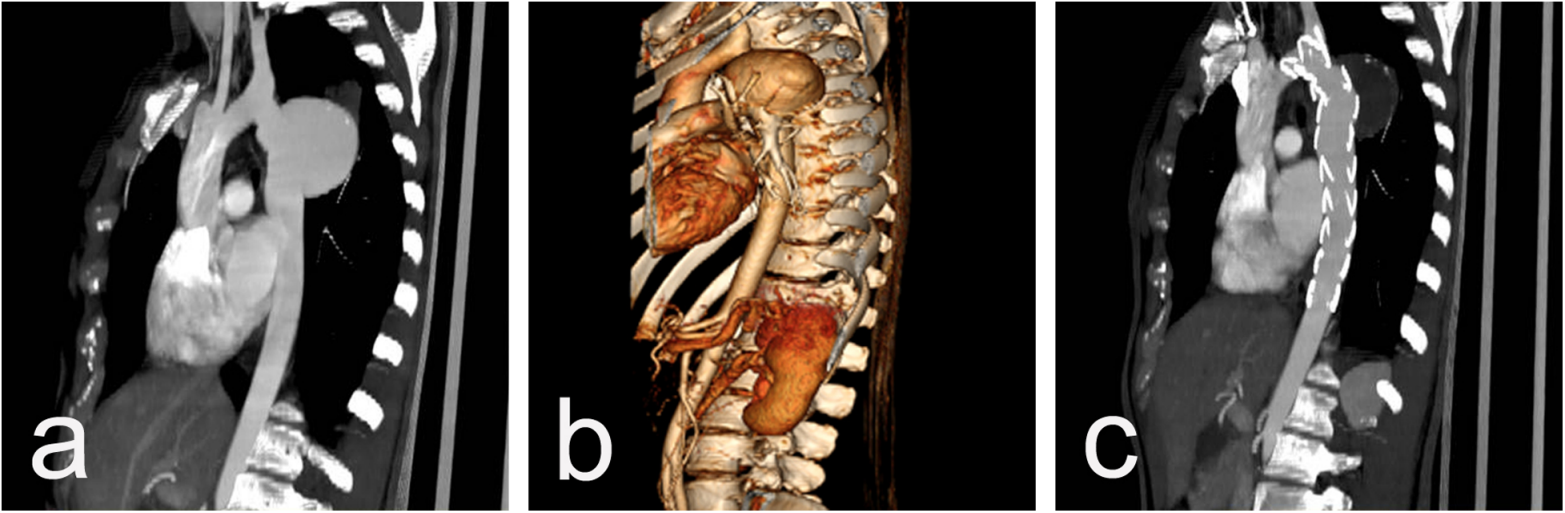
CT scan imaging showing 65 mm post-CoA repair aneurysm in a 41 y/o female, 32 years after surgery **(a,b)** and full cavity thrombosis with significant aneurysm shrinking to 42 mm one year after TEVAR **(c).**

Follow-up: The 30-day and one-year mortality was 0%. The mean follow-up time after stent graft placement was 87 months (range, 23 to 168 months). Seven patients completed five five-years follow-ups and four-ten years follow-up. The survival rate was 80% for 3- and 5-year follow-up. According to data from the CTA performed during the follow-up period, all patients have demonstrated positive aneurysm remodeling with cavity thrombosis and size reduction. Data from the most recent follow-up scan showed that the mean aneurysm diameter had decreased to 54,9 mm (±16,6). (Figures 3a–c) Retrograde dissection from the graft, dissection beneath the implanted graft, and graft displacement were not observed. No endoleaks were diagnosed.

During the follow-up period, rehospitalization required 2 patients. They were admitted for treatment of heart failure. No reinterventions or open surgical interventions were required. Five patients died during the entire follow-up period. The death was non-cardiovascular in one patient (20%), namely liver cirrhosis, while in the other four patients, the death was classified as cardiovascular (80%)- three died because of heart failure, and one presented with aorto-bronchial fistula 61 months after the initial procedure, and despite the subsequent left-sided pneumonectomy, the outcome was lethal. This is the only case that we have classified as an aneurysm-related death.

## 4 Discussion

Left untreated, the CoA has a poor prognosis with a median survival age of 31 years and fatal complications such as aortic rupture, endocarditis, and heart failure (3).

Transcatheter interventions like balloon angioplasty and stent implantation for CoA are feasible treatment choices in particular cases, with stent therapy being a reasonable option for adults with simple, discrete native CoA (10). Concerning children and young adolescents, the interventional approach is associated with a high rate of reinterventions and late aneurysm formation in long-term follow-up (11). Surgical repair of CoA remains a gold standard for treatment in infants and adolescents in order to prevent early or late complications related to proximal hypertension and distal hypoperfusion as the result of the narrowed thoracic aorta (1, 12, 13).

The first surgical CoA repair was performed by Clarence Crafoord in 1944 and represented an end-to-end anastomosis of the aorta of two young adults (12). The surgery is more beneficial at an early age, as soon as possible after diagnosis, due to the anatomical challenges and the high frequency of late complications related to late surgical intervention (6, 14). Open surgical repair has a high success rate and low short-term morbidity and mortality, with benefits concerning improvement in survival, symptom relief, and blood pressure lowering (15, 16). However, despite the evolution of surgical techniques, a significant portion of patients develop late complications, requiring specialized medical follow-up.

Postsurgical aneurysm formation at the site of repair is a potentially life-threatening complication and aortic rupture with a lethal outcome may be the first symptom.

Different pathological abnormalities in the aortic wall of patients with CoA, such as increased stiffness, medial cystic necrosis, and smooth muscle cell rarefaction in the prestenotic segment together with high levels of proinflammatory cytokines, adhesion molecules, and abnormal functional properties of the upper body circulation may persist and lead to modification in aortic wall elasticity and might play a role in the development of post-coarctation repair aneurysm (17, 18). Another possible cause is the damage to the intima during repair and excessive hemodynamic stress on the aortic wall conducted by the rigid patch onto the opposite wall, having a compromised elasticity (19, 20).

Melissano et al. (21) claimed that late aortic aneurysms, regardless of their size, have a worse prognosis than atherosclerotic ones.

Knyshov et al. (22) reported 100% mortality due to hemorrhage in 7–15 years follow-up of untreated patients with post-CoA repairs aneurysm. On the other hand, the redo open surgical repair represents a technical challenge. It is associated with a high mortality rate- up to 14% for in-hospital mortality (7) and a significant percentage of complications as recurrent laryngeal nerve paralysis (13%–36%); bleeding (3.3%–32%) and paraplegia (0.5%–9%) (8, 22, 23).

Endovascular treatment emerged as a safe and effective alternative to surgery, which demonstrated promising potential and results. Nevertheless, some particularities in this population as inherent tortuosity or hypoplasia of the aortic arch, underlying restenosis, proximal to the aneurysm, different mechanical characteristics of the patch, small access vessels, higher likelihood of upper limb claudication after LSA covering in these usually young active patients may be a reason for technical difficulties during endovascular treatment and make the procedure inappropriate (24, 25). The published experience consists of small case numbers but reports excellent results with a procedural success rate of up to 100% and good short and mid-term outcomes concerning survival and positive aortic remodeling (7, 24, 26, 27). Khavandi et al. (28) shared the experience of TEVAR for late post-CoA repair pseudoaneurysms of 17 patients from two UK congenital referral centers. They reported a procedural success rate of 100%, no procedural complications, and four early endoleaks, all resolved by 6 months. For an average follow-up of 31.6 months, they observed aneurysm thrombosis and size reduction; one late no-aneurysm-related death is described. Ince et al. (7) described a series of 6 patients with successful implantation of customized stent-grafts for post-CoA repair aneurysm. They reported uneventful intraprocedural and postprocedural outcomes with no 30-day and 1-year mortality or morbidity and positive aortic remodeling during follow-up. Similarly, Kutty et al. (24) reported no major complications and no late endoleaks, ruptures, conversions to open surgery, or graft migration for a mean follow-up of 24 months in a series of 9 endovascular managed patients, with one reintervention for early endoleak type 1. An international multicenter retrospective study including 74 patients compared the outcomes of endovascular vs. open surgical treatment (21). The authors claimed no differences between the groups regarding early and long-term outcomes with low rates of 30-day mortality, complications, and in-hospital and late reinterventions. They suggested that both surgical and endovascular interventions could be acceptable approaches with favorable early and midterm results in this patient population. However, no RCTs comparing endovascular and open surgical treatment of descending aortic aneurysms exist. Although trials showed improved perioperative and long-term results with TEVAR, concerning aneurysm-related mortality, Goodney et al. (29) stated that the perioperative advantage of endovascular treatment diminished the first year after aneurysm repair, with 5-year survival being even worse after TEVAR vs. open repair (9, 29). Generally, the decision for treatment should be personalized and based on the patient's anatomical and clinical features, and further investigations are mandatory to determine the best treatment option.

We report encouraging results in 12 consecutive patients undergoing endovascular repair for post-CoA repair aneurysms, suggesting it could be a feasible procedure.

According to clinical presentation and CTA data, we defined all cases as complicated, requiring urgent or emergent procedures. All patients experienced symptoms such as chest pain, shortness of breath, or hemoptysis, seven had CTA data for bronchial compression, and one had sustained aortic rupture. The lack of previous routine screening surveillance in the reported group could partially explain the observed larger average aneurysm diameter (67.2 mm), with the largest being 110 mm, compared to other similar series (21, 24, 28) and the fact that the reason for the patient's presentation was the symptoms onset. Stent-graft deployment was save and the procedural success was 100%. Two patients underwent pre-TEVAR open surgical aorto-carotid bypass. In five patients, the endoprosthesis partially or fully covered the LSA after careful evaluation of the vertebral anatomy. No upper limb ischemia, left upper extremity claudication, or subclavian steal syndrome were observed in the periprocedural period or during follow-up. Similarly, Görich et al. (30) concluded that covering the LSA during stent-graft implantation is generally well tolerated and ensures a safe landing zone in a series of patients with no reported significant physical limitation or significant or persistent signs of vertebrobasilar insufficiency during short follow-up. Furthermore, not a single endoleak type II was detected in our group on the postdeployment aortography. Four type I endoleaks were observed; three were treated with additional stent-graft, with good results, and one was left under surveillance and resolved within 6 months. None of our patients experienced severe periprocedural and in-hospital complications. Аccording to the control CTA examinations during follow-up, all patients showed positive remodeling of the aneurysm with complete cavity thrombosis and reduction of the mean aneurysm diameter from 67.2 to 54.9 mm. We observed two early endoleaks type II from LSA, self-resolved by 6 months. Despite the late patient's presentation, the lack of previous surveillance, and the high-risk aneurysm features of the studied group, we report encouraging results concerning mortality and rate of reinterventions, suggesting that TEVAR could be a feasible, safe, and effective treatment of choice in this particular group of patients. The 30-day and one-year mortality rate was 0%. For the mean follow-up period of 87 months, five patients died, with only one having an aneurysm-related lethal outcome. The survival rate for 3 and 5-year follow-up is 80%, and no reinterventions were required, assuming that the whole group underwent regular CT scan examinations.

Based on the patient's baseline characteristics, clinical presentation, and CTA data described, we highlight the alarming necessity of elaboration of routine lifetime surveillance protocol, including regular imaging examinations, modification of risk factors, and blood pressure control for patients with previous CoA repair. All our patients were discharged from surveillance after reaching the age of 18. There is no register or clear standard, established in our healthcare system for the management and follow-up of this usually young group. The malignant course of the post-CoA-repair aneurysm, with the first symptom often being a life-threatening complication, emphasis the need for improvement of awareness among general practitioners, adult cardiologists, patients, and their families for toward early disease recognition and consequently more effective treatment and establishment of clear local guidelines for regular follow-up.

### 4.1 Limitations

This study represents a relatively small sample size. We represent 12 patients treated over two decades and during this period changes were observed in the management and the available devices. Therefore, larger studies would provide more detailed information to confirm our findings. On the other hand of the advancements in surgical techniques for CoA repair over time pose the question would they lead to similar long-term complications, or will their frequency decrease?

## 5 Conclusion

Our experience suggests that endovascular stent-graft implantation is an acceptable treatment of choice for patients with thoracic aneurysms after repair of CoA with appropriate anatomy. We demonstrate high technical success, low immediate, short- and mid-term morbidity and mortality, and low rate of rehospitalizations and reinterventions. Even for patients who have completed longer-term follow-ups, the results are promising. We defined TEVAR in those patients as less invasive than redo surgical repair, a safe and effective procedure. However, the decision on the treatment method should be tailored to each patient and based on the clinical and anatomical particularities. Still, extensive RCTs are needed to determine this approach's usefulness and long-term results. Clear local protocol for routine surveillance of patients with previous CoA repair is mandatory in order to reduce the morbidity and mortality in this usually young patient population.

## Data Availability

The original contributions presented in the study are included in the article/Supplementary Materials, further inquiries can be directed to the corresponding author.
